# Antithrombotic Prophylaxis in the Middle East

**DOI:** 10.4084/MJHID.2011.023

**Published:** 2011-05-24

**Authors:** Samir Arnaout, Hanady R. Samaha, Julien Succar, Imad Bou-Akl, Khaled M. Musallam, Ali T. Taher

**Affiliations:** 1 American University of Beirut Medical Center, Beirut, Lebanon; 2 Saint George Hospital University Medical Center, Beirut Lebanon

## Abstract

Several factors have been proposed to explain the persistence of a high incidence of venous thromboembolism worldwide with its associated morbidity and mortality. Underutilization of anticoagulants and failure of adherence to thromboprophylaxis guidelines are emerging global health concerns. We herein review this alarming observation with special emphasis on the Middle East region. We also discuss strategies that could help control this increasingly reported problem.

## Introduction:

It is well established that venous thromboembolism (VTE) is the most common preventable cause of death in the hospital setting, accounting for 5–10% of fatalities.^1^–^4^ Furthermore, VTE is not restricted to the inpatient setting with several studies showing VTE frequently occurring post hospital discharge.^5^–^7^ Despite the existence of guidelines for VTE prophylaxis in both medical and surgical patients,^8^ medical centers worldwide continue to show elevated incidence rates of VTE.^1^,^9^–^12^ This may be attributed to a significant gap between actual clinical practice and recommendations.^13^–^14^ This review will highlight this alarming problem with emphasis on the Middle East region.

## Results from The ENDORSE Study:

The Epidemiologic International Day for the Evaluation of Patients at Risk for VTE in the Acute Hospital Care Setting (ENDORSE) study was a cross-sectional survey that assessed the adherence to the guidelines across 32 countries in 5 continents.^14^ The study showed considerable variation among countries, with adherence to guidelines ranging from 0.2% to 92% for surgical patients and 3% to 70% for medical patients. The 2004 American College of Chest Physicians (ACCP) guidelines were used to stratify patients for VTE risk and need for anticoagulation.^14^

The compliance with the ACCP guidelines was only assessed in terms of the type of prophylaxis used, with dosing and duration not taken into account because of the differences in dosing recommendations across countries, and because of the cross-sectional nature of the study.^14^ According to the study, the most common VTE risk factors are chronic pulmonary disease and chronic heart failure for medical patients, and obesity for surgical patients.^14^ Complete and partial immobilization (immobilization with bathroom privileges), and admission to an intensive care unit were the most common risk factors for post-discharge VTE in both medical and surgical patients.^14^

Five Middle Eastern countries participated in the study: Egypt, Kuwait, Saudi Arabia, Turkey, and the United Arab Emirates, collectively showing a guideline-adherence rate of 38%.^14^ In comparison, other geographical regions in the study showed the following guideline-adherence rates: America (Brazil, Columbia, Mexico, USA, and Venezuela) 56%, Asia (Bangladesh, India, Pakistan, and Thailand) 9%, Europe (Bulgaria, Czech Republic, France, Germany, Greece, Hungary, Ireland, Poland, Portugal, Romania, Russia, Slovakia, Spain, Switzerland, and the UK) 56%, North Africa (Algeria and Tunisia) and Oceania (Australia) 46% and 57%, respectively. Globally, the study showed that 50% of all patients at-risk were receiving the ACCP recommended prophylaxis.^14^

The ENDORSE study grouped patients according to whether they were medical or surgical cases. Medical patients were stratified based on the medical condition, and surgical patients based on the type of surgery they were undergoing.^14^ It was shown that surgical patients received higher rates of recommended prophylaxis (59%) compared to medical patients (40%). The discrepancy in rates was significant in all regions except the Middle East, with medical and surgical guideline adherence rates being 38% and 39%, respectively (Table1).

Finally, the ENDORSE study also reported the percentages of patients that received some form of prophylaxis irrespective of whether it followed the risk-stratification guideline of the ACCP. In the Middle East region, 45% of patients received some form of prophylaxis (41% medical, 48% surgical), with the other regions performing as follows: America 67% (63% medical, 71% surgical), Asia 11% (14% medical, 9% surgical), Europe 62% (49% medical, 71% surgical), North Africa 48% (31% medical, 72% surgical), and Oceania 66% (51% medical, 82% surgical).^14^

## Results from the AVAIL ME Study:

The Assessment for VTE Management in Hospitals in the Middle East (AVAIL ME) study was the first comprehensive evaluation of VTE prophylaxis in the Middle East region. It was conducted to properly evaluate the status of anticoagulation practices in the Middle East and to serve as a foundation for attempts at quality improvement.^15^ The study included countries from the Middle East (Kingdom of Saudi Arabia, Lebanon, Syria, and United Arab Emirates), and from Central Asia (Georgia, Iran, and Kazakhstan).^15^ Previous studies on VTE prophylaxis in the Middle East were performed on diverse populations, across different timelines, employed varied methodologies, and were limited to predefined specialties (orthopedics, gynecology, etc), which made them unsuitable for adequate comparison.^15^

The study showed consistently lower rates of adherence to the guidelines in the Middle East region compared to global figures, thereby confirming the findings of the ENDORSE study.^15^ Moreover, the study underlined the inappropriate utilization of prophylaxis in the Middle East in patients with contraindications, further emphasizing the divergence from the guidelines compared to the other regions.^16^

The study showed that 54.9% of patients received drug prophylaxis for VTE, but that only 36.9% received the 2004 ACCP recommended prophylaxis according to risk (the most up-to-date guidelines at the time of data collection).^15^ Of the 2,266 patients in the study, 82.9% were eligible for prophylaxis according to the guidelines. 51.2% obtained some form of VTE prophylaxis, but only 37.8% according to the ACCP. 50.1% of eligible patients received drug prophylaxis (90.2% low molecular weight heparin, 10.7% unfractionated heparin, 1.5% vitamin K antagonists, and 0.1% fondaparinux), 16.4% received mechanical prophylaxis (graduated compression stockings in 97% of cases), 15.3% received both modalities, and the remaining received no prophylaxis.^15^

Stratification into risk groups revealed that guideline application was observed in 86.3%, 41.1%, 48.3%, and 24.5% in the low, moderate, high, and very high-risk groups respectively. Concurrently, some form of VTE prophylaxis was given to 17.9% of the low-risk patients, and in 41.7%, 60.6%, and 66.9% of the moderate, high, and very high-risk groups, respectively.^15^

The Kappa coefficient was calculated to evaluate concordance between eligibility for VTE prophylaxis and drug application, both according to guidelines, and was found to be equal to 0.16 (it ranges between 0 = no concordance, and 1 = perfect concordance) with a statistically significant *P* < 0.001. The study revealed that 45.1% of patients who should have received prophylaxis did not, and 26.9% of patients who were not eligible for prophylaxis did receive it. Separate Kappa values were also calculated after risk-stratification with values of 0.09 for moderate-risk, 0.20 for high-risk, and 0.09 for very high-risk patients (all statistically significant). Concordance for low-risk patients cannot be calculated because they should not receive prophylaxis.^15^

In the same study, it was observed that there were significant differences in adherence depending on the medical condition, with 40.2% of patients with ischemic stroke, 38.8% of cancer patients, and 35.7% of patients with heart failure (NYHA class III or IV) receiving prophylaxis according to the guidelines compared to only 26.7% of patients with hemorrhagic stroke and 26.8% of patients with renal failure^15^. This discrepancy in the use of prophylaxis among different medical conditions was also found in a previous study in Lebanon which showed higher prophylaxis rates in intensive care unit patients (62.5%) compared to patients admitted for malignancies (17.7%).^17^

In addition to the AVAIL ME study, several studies continue to show discordance in the Middle East between VTE prophylaxis and the guidelines.^18^–^19^ These figures are more pronounced given the elevated proportion of the high-risk group in the region.^15^ The observation that medical patients in the Middle East region receive less drug prevention than surgical patients is congruent with the results of other regional studies.^20^–^23^ These results continue to emerge despite the finding that more than 70% of VTE events occur in medical patients compared to only 30% or less in surgical patients.^12^,^24^–^25^

## Towards More than an Ounce of Prevention:

Inappropriate thromboprophylaxis is a substantial problem that requires immediate attention. Efforts should be made to systematically assess patients at risk for VTE using practical models, urgently implement hospital-wide strategies, and provide appropriate prophylaxis to prevent this common and avoidable disease. This remains essential in developing countries with limited health care resources. Educational sessions targeting practicing physicians and staff and efforts towards changing guidelines to hospital policy should also ensue.

Several VTE risk assessment models (RAMs) and algorithms have been suggested.^20^,^26^–^31^ However, most of these RAMs have not been prospectively validated, and the only two validated models present limitations that preclude widespread implementation.^30^–^31^ The adoption of electronic tools was found to be effective in encouraging physicians to use prophylaxis at least amongst subgroups of patients at high-risk of thrombotic complications.^30^–^31^ The electronic alerting systems, however, require sophisticated technology infrastructure and considerable financial resources, and are thus unlikely to find widespread acceptance across the many institutions that admit patients at risk of thrombosis. Self-explanatory, easy, suitable and effective RAMs may have the potential to help physicians manage their patients without the need for supplementary electronic tools, and may result in an increasing implementation of antithrombotic prophylaxis, in compliance with the recommendations of the main international guidelines and with the auspices of several world health organizations. A new simple points score system, the Padua Prediction Score,^32^ was recently reported. The system was evaluated for its potential to detect hospitalized medical patients at high-risk for developing VTE, and its value was prospectively assessed in a broad spectrum of consecutive patients admitted to an Internal Medicine ward in a two-year period. All patients included in the study were prospectively followed-up for up to three months after admission in order to assess the incidence of symptomatic VTE. The simple 20-point RAM adopted clearly discriminated between hospitalized medical patients at high- and low-risk of VTE complications. The implementation of in-hospital thromboprophylaxis in patients classified as being at high-risk of thrombosis according to this RAM was highly effective, and was associated with an acceptably low-risk of bleeding. However, this benefit of this model needs to be prospectively evaluated.

Following pilot results of the AVAIL ME study, the alarming gap in adherence to practice guidelines led most involved centres, and many others, to start initiatives of careful planning to control the problem. Educational sessions targeting practicing physicians and staff and efforts towards changing guidelines to hospital policy ensued. These efforts are to be prospectively evaluated to determine their impact on the problem currently at hand.

## Conclusions:

The Middle East region has consistently lower rates of adherence to VTE prophylaxis guidelines, in both medical and surgical populations, compared to other regions. These shortcomings need to be addressed with outmost importance given the preventable nature of VTE, and the potential to substantially decrease associated morbidity and mortality in the region.

## Figures and Tables

**Table 1.** Adherence to guidelines in medical and surgical patients across regions evaluated by the ENDORSE study.

Region	Medical	Surgical
America	49%	63%
Asia	12%	7%
Europe	41%	67%
Middle East	38%	39%
North Africa	28%	71%
Oceania	42%	72%
